# Ethical Issues in Providing Online Psychotherapeutic Interventions

**DOI:** 10.2196/jmir.2.1.e5

**Published:** 2000-03-31

**Authors:** Craig A Childress

**Keywords:** Internet, Psychotherapy, Psychiatry, Ethics, Quality of Health Care, Remote Consultation, Physician-Patient Relations, Professional-Patient Relations, Teleadvice, Electronic Mail

## Abstract

The Internet offers psychotherapists a new communication medium through which they can deliver psychotherapeutic interventions that are appropriate to the medium. Yet online psychotherapy also offers new ethical challenges for therapists interested in providing online psychotherapeutic services. The differences between interactive text-based communication and in-person verbal communication create new ethical challenges not previously encountered in face-to-face therapy. This article will examine the Internet's potential for providing online psychotherapeutic interventions and will review the ethical issues involved with providing interactive text-based psychotherapy.

## Introduction

The Internet provides a new medium for interpersonal communication that holds the potential for delivering forms of psychotherapeutic interventions that are appropriate to the medium. The challenges facing psychotherapists lie in discovering what types of interventions are appropriate to this new medium and in delineating the potential advantages and limitations inherent to this new communication format.

Mental heath professionals are already exploring the usefulness of the Internet medium in delivering online psychotherapeutic interventions [1-3], and several professional associations have developed general guidelines for the online delivery of therapeutic services [4-6]. Simply practicing within recommended guidelines, however, does not release each individual therapist from the personal responsibility to be aware of, and to independently evaluate, the variety of ethical issues involved in the practice of online therapy. The obligation to act ethically cannot be transferred to an organization, but remains the personal responsibility of each therapist seeking to practice online.

The Internet provides several different communication systems, some of which are similar to in-person communication (e.g. video technology), some of which are similar to traditional print media (e.g. web pages), some are hybrids of interactive in-person communication and traditional written-word communication (e.g. email and chat), and some are unique to the Internet medium (e.g. MOOs and MUDS). The ethical and pragmatic challenges facing psychotherapists seeking to use the Internet to deliver online psychotherapeutic interventions will vary depending upon which communication medium is being used.

This article will focus primarily on the use of email to deliver online psychotherapeutic interventions. While two-way video technology may someday become widely available, it is unclear whether it will ever gain acceptance as a common means of personal communication on the Internet. Two-way video technology has long been available for telephones, but people have not rushed out to buy video telephones. It is also questionable whether people will feel comfortable having video cameras in their homes. Cameras attached to personal computers may be viewed as an unwelcome intrusion into personal privacy. While interactive real-time video communication holds potential in a variety of health related interventions, the technology may remain limited to large organizational and hospital uses, without widespread dissemination into personal use. Should two-way interactive video become widely accepted and available, then it can be incorporated into delivering therapeutic interventions; and since video is a form of "-to-face" interaction, the ethical issues will, to a large extent, be similar to those encountered with in-person, face-to-face therapy.

Currently, however, email provides the backbone of interactive online communication, and it may hold the greatest potential for delivering psychotherapeutic interventions using the Internet [7]. The most significant feature of email is that it is text-based communication, and this is the source of its greatest strengths and its greatest limitations.

## Issues Specific to Email Psychotherapeutic Interventions

As text-based communication, email is asynchronous (not in real-time), which allows the participants to communicate at their own convenience. The asynchronous nature of email can facilitate the client's perception of the therapist's availability, and may provide the client with a more intense psychological holding environment [8] than is available through a traditional in-person relationship. The perceived availability of the therapist may also enhance the client's ability to incorporate the therapist's presence into daily life. Rather than waiting for the weekly in-person session to discuss an issue, the client can instead write the therapist an email while the issue is still active, thereby evoking the therapist's psychological presence in the moment.

Asynchronous text-based communication also allows the parties involved to carefully consider and edit their communication, which is an advantage over real-time text or in-person communication. Even asynchronous video and audio communication do not offer the advantages of editing afforded by interactive text-communication. Asynchronous text communication actually involves writing small-scale essays, similar to traditional letter writing. The interactive nature of email communication comes from the speed by which these essays or letters are exchanged, which gives the illusion of greater interactivity than might actually be present.

Interactive text-based communication also involves the loss of the non-verbal social cues that provide valuable contextual information in conversation and can influence the interpretation of meaning in communication. Miscommunication may therefore be more likely with interactive email communication. The loss of physical social cues may also increase the client's tendency to project personal psychological material onto the blankness of cyberspace communication. This enhanced tendency toward projection of personal material in text-based communication may be helpful in some forms of psychotherapeutic interventions, and it may offer distinct advantages over in-person communication as well as a potential risk for increased miscommunication.

Nor does email need to be the sole intervention offered to clients. Email might be fruitfully integrated into a traditional in-person psychotherapy, allowing the client to continue the therapeutic process between in-person sessions with the therapist. The in-person therapist might prescribe certain writing assignments to the client, which can then be emailed to the therapist between sessions. The issues raised in the email can then be addressed during the in-person session. There is some evidence that persons may feel more comfortable self-disclosing through a computer [9]; and clients might use email communication to broach sensitive issues, such as past experiences of abuse, that they may be unwilling to address during the in-person session. Having once raised the issue in email, it can then be dealt with more extensively during the in-person sessions.

Therapists might also incorporate email into cognitive/behavioral interventions. The in-person therapist can prescribe homework involving the monitoring of behavior or thoughts. This daily homework could then be emailed to the therapist, allowing the therapist more timely access to the client's progress. Daily emailing of homework to the therapist might also help motivate and reinforce the client's completion of the assigned homework on a daily basis. It would also allow therapists to monitor the intervention throughout the week; and if corrections to the behavior program are needed, they can be developed and incorporated into the program prior to the next in-person session. Incorporating email into a traditional in-person therapy holds the potential to increase the speed of therapeutic progress, the depth of material discussed, and cost-effectiveness of treatment [10].

Email clearly holds potential for delivering psychotherapeutic interventions, either by itself or in conjunction with a traditional in-person relationship. This potential has led increasing numbers of psychotherapists to begin exploring the use of email (and chat) to deliver psychotherapeutic interventions [1]. However, the use of a new communication medium involving interactive text-based communication raises unique ethical questions not previously addressed within the confines of the in-person therapeutic relationship. The delivery of therapeutic interventions solely through the Internet (i.e., not in association with an in-person therapeutic relationship) offers the most problematic ethical issues, and it will be those issues that will be focused on in the remainder of this article.

## Guiding Ethical Principles

### Ethical Responsibility to Provide Service

One of the initial ethical issues involves the responsibility of mental health professionals to provide services to meet the demand of consumers. While reservations may exist regarding the provision of online psychotherapeutic services, if consumers desire such services and if there is a reasonable expectation that online therapeutic interventions can be beneficial, then we have a professional obligation to address this demand. If mental health professionals do not step forward to provide these services, then consumers will be forced by the lack of response from the professional community to seek online therapeutic services from unlicensed and untrained providers. Inasmuch as there appears to be a reasonable expectation that some form of psychotherapeutic intervention can be developed that can appropriately be delivered using Internet text-based communication [7,11], it is incumbent upon the field of professional psychology to explore the ethical and professionally responsible delivery of online psychotherapeutic interventions. Yet, once we accept the obligation to explore the professional delivery of online psychotherapeutic services, then other problematic ethical issues emerge [12-15].

### Do No Harm

The evaluation of the potential harms associated with any treatment intervention needs to be considered within the context of the potential benefits to be accrued from the intervention [16]. Only by considering both the potential risks and the possible benefits can we appropriately evaluate a proposed intervention. The simple presence of risk does not necessarily preclude the use of an intervention if it is sufficiently justified by the potential benefits. With interventions that have a reasonable likelihood of being beneficial for the client, the important issue becomes for the therapist to understand the nature of the risks, to minimize the risks to the extent possible, to fully inform clients as to the nature of the risks within the context of the possible benefits, and then to allow the clients to make an informed decision regarding their treatment options.

In assessing the risks of online psychotherapy it is also important to note that in-person psychotherapy is not without risks. In-person clients can become sexually attracted to the therapist and vice versa; therapists can be incompetent in the delivery of services; the therapist's confidential records are vulnerable to being stolen or viewed by unauthorized persons even if stored in a locked office and a locked file cabinet; miscommunication can occur during in-person therapy; and clients receiving in-person therapy can deceive and mislead the therapist. The issues with the delivery of online psychotherapy (e-therapy) are the extent to which traditional risks are enhanced in text-based communication, the possible emergence of novel types of risks not present in face-to-face therapy, and the degree to which the potential benefits justify the possible risks.

The use of email to deliver therapeutic interventions opens several areas of potential risk to online consumers. Clients of online services can be at greater risk for breaches in confidentiality [16,17]. This increased risk to confidentiality occurs at the therapist's end, at the client's end, in the transmission of information, and in the potential for legal subpoena of records. Therapists using the Internet to deliver therapeutic interventions should evaluate the security of their websites and computers against outside intrusions that would compromise client confidentiality. These intrusions might include high-tech invasions by hackers downloading files from the therapist's computer, to low-tech intrusions involving the inappropriate availability of the client's email to the therapist's office staff or family members. Therapists using the Internet to deliver online psychotherapeutic interventions may wish to consider installing systems which use firewalls, passwords, and backup data storage systems to increase the security of email communications and to protect against the inadvertent loss of clinical files resulting from computer malfunctions.

Online consumers of mental health services must likewise consider security issues on their end of the communication. Other persons who have access to the client's email, such as employers or family members, may be able to read stored copies of the client's email or incoming email from the therapist. Additionally, human error in addressing email has sometimes resulted in email being sent to the wrong person. Inadvertently sending private information meant for the therapist to a friend or family member can result in embarrassing and painful situations for the client. Potential online consumers of mental health interventions need to be informed about these potential breaches of confidentiality in order to fully evaluate the possible risks versus the potential benefits of online psychotherapy.

Breaches of confidentiality can also occur as email is in transit. The potential vulnerability of email in transit may not, however, accurately represent its actual vulnerability in practice. While email may be intercepted in transit, it is unlikely that individual emails sent between private parties are actually intercepted and read from the incredible volume of email sent each day. Still, this potential breach in confidentiality needs to be understood and evaluated by clients before choosing to engage in online psychotherapy. Encryption technology can improve security of email communication, and online therapists may wish to make encryption of email routinely available to their clients.

Online mental health clients also need to consider the possibility that email records may be subject to subpoena. While professional communication with physicians and attorneys is considered legally privileged, it is unclear if this legal protection extends to psychotherapists. The standards for recognition of legal protection of privileged communication may also vary from one jurisdiction to another. Online psychotherapists should consider their policy regarding the disclosure of records in response to legal subpoena, and clients need to be informed about this possible breach of confidentiality.

The use of email to provide psychotherapeutic interventions also entails other risks to clients beyond those associated with the confidentiality of communication. For example, the loss of nonverbal cues significantly impedes the therapist's ability to make a full assessment and diagnosis. Important in-person cues, such as flattened or inappropriate affect, characteristics of speech, memory function, or physical evidence of a medical condition that may be associated with the psychological symptoms, are all lost in email communication. An impaired ability to make an adequate diagnosis will adversely affect the ability of online therapists to develop appropriate treatment plans and, as a result, the treatment interventions that are developed may be to the detriment of the client. Online testing may improve diagnostic capabilities [18], and gathering a full psychosocial history may be facilitated by online questionnaires; yet the loss of visual and auditory cues will still affect the therapist's diagnostic ability, and the impact of this diminished diagnostic capability needs to be carefully considered. Still, while problems making online diagnoses may limit the scope of issues appropriately addressed in online therapy, some types of online interventions, such as interactive journaling [7] or humanistic/existential approaches, may nevertheless be developed that are appropriate for delivery in a text-based format with some populations.

The increased potential for miscommunication in text-based therapy may also increase the risk of inadvertently harming clients and perhaps re-traumatizing emotional injuries disclosed during the course of online therapy. Text-based interactive communication is more vulnerable to miscommunication because it lacks the non-verbal cues associated with in-person communication that modify meaning and provide context for the interpretation of meaning. Furthermore, interactive text communication is not the normal means of interpersonal communication for most therapists trained in in-person psychotherapy. Therapists may therefore lack the writing skills needed to express subtleties of meaning through the written word.

Working with psychological issues typically involves addressing conflicting client motivations involving a desire for self-disclosure aimed at securing help for painful personal issues, and competing motivations directed toward maintaining interpersonal defenses to preserve self-esteem and prevent re-traumatization of emotional injuries. Interactive text-based communication often sounds harsher than intended. Online miscommunication may result in clients feeling hurt because they perceive the therapist's response as being critical or rejecting. Online clients also do not have the benefit of the interpersonal holding environment offered by the in-person relationship in which to interpret and integrate the therapist's comments, and injured online clients may be more likely to simply withdraw from the relationship into the blankness of cyberspace, taking their injury with them. Since nonverbal feedback cues that might signal the miscommunication, such as the client's body language and facial expression, are not available in email communication, online therapists may often be unaware of the miscommunication and therefore will be unable to address the client's injury.

This possibility of emotional injury and re-traumatization may be further exacerbated by the increased self-disclosure and disinhibition associated with online communication [9,19]. While increased self-disclosure may be helpful in some therapy circumstances, it may also involve clients prematurely moving past defenses designed to protect them against emotional injury and re-traumatization. This may leave them more vulnerable to injury should they interpret a therapist's communication as being critical or rejecting.

Clients in online psychotherapy may also be at increased risk of harm if the online intervention is not effective in creating change in the client's life, yet offers enough solace so as to reduce the client's motivation to seek more beneficial in-person therapy. Consumers of online mental health services are at risk in this case not because of a direct effect of the online intervention, but because the online intervention prevents them from seeking treatments that will more effectively address their needs. However, e-therapy may also serve as a convenient and helpful entry into the mental health system for many persons who might benefit from therapy but who are reluctant to begin in-person therapy because of the social stigma associated with psychotherapy, their anxiety of addressing emotional issues, and the physical inconveniences of scheduling in-person therapy sessions. For such persons, the convenience and perceived anonymity associated with computer-mediated communication may encourage them to contact an online psychotherapist. Their initial online therapeutic relationship may help demystify psychotherapy and facilitate their entry into in-person mental health treatment.

The ethical practice of e-therapy requires that therapists develop a thorough understanding of all of these issues. Online discussion groups dealing with Internet psychology can help therapists explore some of these issues. Yet, despite the therapist's professional evaluation of the issues involved with providing online therapeutic interventions, the ultimate issue is the degree to which the potential benefits justify the possible risks, and a decision on this issue can only come from a fully informed client. While mental health professionals can decide that the potential benefits associated with the intervention do not justify the risks, the opposite decision, that the benefits do justify the risks, can only be made by a fully informed client. Therapists seeking to provide online psychotherapeutic interventions must, therefore, be informed as to the potential risks so that they can take every possible precaution to reduce or eliminate those risks, and so that they can fully educate potential clients regarding the possible risks associated with e-therapy.

### Providing Effective Interventions

While controversies exist as to what criteria should be used to evaluate the effectiveness of psychotherapy, in-person psychotherapy nevertheless has an extensive history and well-elaborated theoretical frameworks supporting its use. Both history and theoretical frameworks are missing from the practice of interactive text-based therapy, and it is currently unclear to what degree traditional therapeutic orientations and models can be translated into online, text-based communication. Most psychotherapy depends, to a greater or lesser degree, on the development of the therapeutic relationship [20-22]. However, it is precisely the nature of the therapeutic relationship that is most impacted by text-based communication.

The ethical practice of e-therapy requires the therapist to have a clearly delineated model of psychotherapy appropriate to delivery in a text-based format [7,18]. In the emerging field of online psychotherapy, it would also behoove the ethical practice of e-therapy if therapists remained close to empirically derived support for the interventions used until more experience is gained with regard to the medium of interactive text-based communication. Therapists providing online psychotherapeutic interventions should also contribute to the developing understanding of e-therapy by conducting quantitative and qualitative evaluations of the services they deliver.

### Practicing Beyond the Boundaries of Competence

For psychologists, the Ethical Code of the American Psychological Association [23] specifically directs that psychologists should practice only within the area of their competence based on training and experience (Standard 1.04a); and that where standards for training do not yet exist, psychologists should " reasonable steps to ensure the competence of their work and to protect patients, clients, students, research participants, and others from harm" (Standard 1.04c; p. 1600). Psychotherapists trained in traditional psychotherapy need to carefully consider whether they are competent to practice in an interactive text-based format, and to evaluate by what manner and training they achieved their competence in this new communication medium.

Interactive text-based communication offers an entirely new communication format that differs significantly from in-person verbal communication. The nonverbal cues that in face-to-face communication provide valuable information that modifies meaning and aids interpretation of the communication are significantly absent in text-based communication. It takes considerable skill to communicate emotion and contextual intent solely through the written word. Text can often sound harsher than intended and, without contextual cues such as tone of voice and body language, text-based communication is more likely to be misunderstood and misinterpreted. With text-based communication there is also a greater likelihood of projective psychological material emerging in the absence of the physical presence which serves to ground in-person communication. Skill in verbal communication does not necessarily translate into skill in written communication, especially interactive text-based communication that involves a series of interpersonal interpretations within each exchange.

Without clearly delineated models for text-based psychotherapy, and without training in the subtleties of interactive written-word communication, therapists seeking to provide online psychotherapy need to carefully evaluate their current level of competence to practice in a text-based format.

### Professional Accountability and the Redress of Grievances

Mental health professionals wishing to practice online also need to consider their legal authority to practice in a jurisdiction in which they are not licensed to practice [24]. This issue extends beyond the legality of their activity to include the rights of clients to redress grievances.

The ethical practice of online psychotherapy must provide for the client's ability to redress grievances. Clients should be clearly informed prior to beginning an online therapeutic relationship about the regulatory agencies and professional associations governing the therapist's work [5]. Still, simply being informed about oversight agencies may not offer online clients an actual ability to redress grievances when the therapist and client may live in separate jurisdictions separated by hundreds or even thousands of miles [24]. For example, while it may be possible for a client in India to file charges with an ethics board or Attorney General located in the therapist's home jurisdiction of Wisconsin, the practical limitations imposed by distance and the financial resources needed to overcome such limitations may leave the client unprotected in fact.

Laws governing the appropriate practice of psychotherapy, such as ordinances governing the release from confidentiality to report child abuse, may also differ from one geographic jurisdiction to another. When the therapist and client live in two different legal jurisdictions with differing laws regarding the practice of psychotherapy, which jurisdiction's laws take precedence and govern the client-therapist relationship?

In order to avoid the many problematic legal and professional issues related to practicing psychotherapy online, some therapists may be tempted to define their online work as psychoeducational rather than psychotherapeutic. While some online work can legitimately claim to be primarily educational, therapists treating individual clients across multiple sessions should carefully consider whether their work is primarily educational or therapeutic. One of the central issues in making this distinction is whether the client perceives an individual professional relationship has been established. While it may be tempting to try and circumvent legal and professional liability for online work by defining it as psychoeducational, it creates significant ethical problems if such a definition misrepresents the service. Ethical problems can also arise if the online service being provided is held out as therapeutic on one web page, with a disclaimer of psychoeducational intent located on a separate web page. An ethically appropriate description of the online service must clearly, consistently, and accurately describe the intent of the service and the nature of the professional relationship involved.

### Informed Consent

The absence of physical presence also impacts the ability to verify identity. Without the ability to verify identity the issue of treating minors without parental consent becomes problematic. Therapists seeking to practice online must evaluate what steps will be taken to verify the age of clients so as to not treat minors without the knowledge and consent of their parents.

In addition, the issue of informed consent is closely related to the issue of disclosure. As discussed earlier, in order to make an informed consent to treatment clients need to fully understand the potential risks and benefits associated with an intervention. Specific risks that clients need to be informed about involve the possibility that inadvertent breaches of confidentiality may occur with online communication, the experimental nature of online psychotherapeutic interventions and the possibility of unknown and unintended consequences, and the potential for miscommunication in text-based communication [25].

In some ways, however, the Internet offers advantages in developing an informed consent process. Professional web pages allow for multi-faceted and multi-layered discussion of relevant issues which remain constantly available on the Internet for clients to review. Web pages can address issues such as the potential risks involved with online treatment and the theoretical underpinnings of the treatment. The discussion of informed consent through email also allows for a documented record of the informed consent process.

### Crisis Intervention Planning

Online psychotherapists need to consider plans for addressing the variety of crises that may present in therapy including suicidal clients, physical and sexual abuse, threats to harm others, and the possible discovery that the client's issues would more appropriately be addressed with intensive in-person therapy or hospitalization. Prior to beginning a therapeutic online relationship, therapists may wish to discuss crisis plans and develop in-person referrals local to the client in preparation for possible future crises. Such crisis planning should include obtaining a verified valid street address and phone number that would allow the therapist to invoke the local police should such an intervention become indicated.

### Boundary Issues

Therapists interested in providing online interventions also need to consider the possible boundary issues involved with establishing an online therapeutic relationship. For example, with instant message systems clients might be alerted every time the therapist is online and could send the therapist instant messages for chats every time the therapist signs onto the Internet. Clients might also access the therapist's personal web page or sign onto online discussion groups to which the therapist also belongs. In addition, some clients may continue to send the therapist emails after the termination of the relationship, and e-therapists will need to consider their response to such ongoing contact. Some clients may also use the Internet to harass or stalk current or former therapists.

## Conclusions

The Internet provides new opportunities to provide beneficial psychotherapeutic interventions with clients. Yet in providing online psychotherapeutic interventions, therapists need to evaluate the degree to which the online clients are informed regarding the potential risks they are assuming, including the risk that because there is little formal research on the process of online therapy, there may arise unforeseen and unanticipated problems. Therapists also need to evaluate their own competency to deliver text-based interventions and the source of this competency in their background and training. Before providing online therapy, mental health professionals also need to develop theoretical models for the interventions being used that are appropriate to delivery in a text-based format.

Therapists seeking to provide online interventions also need to become thoroughly familiar with the risks associated with e-therapy and with the professional guidelines being developed for the ethical practice of e-therapy. Online professional discussion groups devoted to Internet psychology may help by offering professional consultation regarding issues related to e-therapy; yet therapists cannot rely entirely on professional guidelines or online consultation, and must actively accept their personal responsibility for fully understanding, considering, and addressing the potential ethical issues involved with online therapy.
